# Mediating Retinal Ganglion Cell Spike Rates Using High-Frequency Electrical Stimulation

**DOI:** 10.3389/fnins.2019.00413

**Published:** 2019-04-30

**Authors:** Tianruo Guo, David Tsai, Chih Yu Yang, Amr Al Abed, Perry Twyford, Shelley I. Fried, John W. Morley, Gregg J. Suaning, Socrates Dokos, Nigel H. Lovell

**Affiliations:** ^1^Graduate School of Biomedical Engineering, UNSW Sydney, Sydney, NSW, Australia; ^2^Department of Biological Sciences, Columbia University, New York, NY, United States; ^3^Department of Electrical Engineering, Columbia University, New York, NY, United States; ^4^VA Boston Healthcare System, Boston, MA, United States; ^5^Department of Neurosurgery, Massachusetts General Hospital and Harvard Medical School, Boston, MA, United States; ^6^School of Medicine, Western Sydney University, Penrith, NSW, Australia; ^7^School of Biomedical Engineering, The University of Sydney, Sydney, NSW, Australia

**Keywords:** neuromodulation, retinal ganglion cell, high-frequency electrical stimulation, retinal implant, computational modeling, *in vitro* patch-clamp

## Abstract

Recent retinal studies have directed more attention to sophisticated stimulation strategies based on high-frequency (>1.0 kHz) electrical stimulation (HFS). In these studies, each retinal ganglion cell (RGC) type demonstrated a characteristic stimulus-strength-dependent response to HFS, offering the intriguing possibility of focally targeting retinal neurons to provide useful visual information by retinal prosthetics. Ionic mechanisms are known to affect the responses of electrogenic cells during electrical stimulation. However, how these mechanisms affect RGC responses is not well understood at present, particularly when applying HFS. Here, we investigate this issue via an *in silico* model of the RGC. We calibrate and validate the model using an *in vitro* retinal preparation. An RGC model based on accurate biophysics and realistic representation of cell morphology, was used to investigate how RGCs respond to HFS. The model was able to closely replicate the stimulus-strength-dependent suppression of RGC action potentials observed experimentally. Our results suggest that spike inhibition during HFS is due to local membrane hyperpolarization caused by outward membrane currents near the stimulus electrode. In addition, the extent of HFS-induced inhibition can be largely altered by the intrinsic properties of the inward sodium current. Finally, stimulus-strength-dependent suppression can be modulated by a wide range of stimulation frequencies, under generalized electrode placement conditions. *In vitro* experiments verified the computational modeling data. This modeling and experimental approach can be extended to further our understanding on the effects of novel stimulus strategies by simulating RGC stimulus-response profiles over a wider range of stimulation frequencies and electrode locations than have previously been explored.

## Introduction

Extracellular electrical stimulation is extensively used in electro-neural interfaces for the central and peripheral nervous systems (Gybels, 1981; Deep-Brain Stimulation for Parkinson’s Disease Study Group et al., 2001; Kilgore and Bhadra, 2004; Guenther et al., 2012). In particular, retinal prosthesis aims to restore functional visual percepts to those suffering from retinal degenerative diseases, by electrically stimulating the surviving neural tissue of the retina (Rizzo and Wyatt, 1997; Palanker et al., 2005; Weiland et al., 2005). In such cases, the aim is to elicit visual percepts by activating the remaining retinal neuronal populations in a controlled spatiotemporal pattern.

Considerable research into high-frequency (defined as being higher than 1.0 kHz) electrical stimulation (HFS) is underway to understand the extent to which neuronal activity can be quantitatively controlled with greater spatiotemporal precision, in order to improve the performance of neuroprostheses. For example, HFS ranging from 2.0 to 20 kHz has been extensively used to block unwanted or unregulated generation of nerve impulses in many disabling conditions (Kilgore and Bhadra, 2004). In addition, the effects of a large range of stimulation frequencies (5–50 kHz) have been investigated to selectively block different types of peripheral nerve fibers (Joseph and Butera, 2011). HFS up to 5 kHz was also reported to generate more stochastic firing in auditory nerve fibers (Litvak et al., 2001, 2003). Finally, recent clinical studies have also begun to assess how HFS might affect the efficacy of neural implants for the cochlear (up to 2.4 kHz) (McKay et al., 2013), the retina (up to 3.33 kHz) (Horsager et al., 2009), and the spinal cord (up to 10 kHz) (Tiede et al., 2013; Van Buyten et al., 2013).

In the retina, recent studies suggest that epiretinal HFS (1.0–6.25 kHz) is able to differentially activate functionally-different retinal ganglion cell (RGC) types (Cai et al., 2013; Twyford et al., 2014; Kameneva et al., 2016; Guo et al., 2018b). The RGC types examined demonstrated a characteristic non-monotonic, stimulus-strength-dependent response during HFS, offering the intriguing possibility of targeting certain functionally-distinct RGC types without simultaneously producing any significant response in other types. Given the promising performances of HFS in retinal and other functional electrical stimulation regimes, it is important to explore the precise mechanisms underlying HFS-induced strength-dependent activation. “What is the main intrinsic property that dominates the response of RGCs to biphasic HFS? and how HFS-induced strength-dependent activation can be modulated across a wide range of stimulus frequencies?”

In answering these questions, we began with *in silico* investigations to gain insights into the mechanisms underlying experimentally-observed non-monotonic stimulus-response profiles during HFS. The model included accurate 3D morphological reconstruction of a single RGC, and its electrical response behavior was optimized against multiple whole-cell recordings from the same cell for accurate biophysics (Guo et al., 2016). Using this model, we identified a correlation between RGC response patterns during HFS and RGC intrinsic properties, in order to elucidate the likely mechanisms underlying neuronal excitation by extracellularly applied HFS. In addition, RGC stimulus-strength-dependent properties over a wide range of stimulation frequencies ranging from 1.0 to 9.0 kHz, were predicted using the computational model. In the second stage, we performed *in vitro* experimentation to verify the mechanisms and results predicted by the computational modeling, for a generalized stimulus electrode placement without a priori knowledge of axon initial segment (AIS) location, a limitation of previous HFS work on RGCs (Cai et al., 2011, 2013; Twyford et al., 2014).

## Materials and Methods

### Morphologically-Realistic and Biophysically-Accurate RGC Model

The RGC model was implemented using the NEURON computational software (Hines and Carnevale, 1997). In order to reconstruct the 3D cellular morphology, an OFF RGC was filled with neurobiotin-Cl using a whole-cell patch pipette. The retina was subsequently fixed in paraformaldehyde, with the filled cell reacted against Streptavidin–Alexa 488, as described previously (Tsai et al., 2012; Guo et al., 2016). The behavior of this RGC model closely replicated published experimental RGC responses to epiretinal electrical stimulation (Twyford et al., 2014; Guo et al., 2018b). Detailed anatomical information of the neuron was included in the model. A compartmentalized axon of 0.94-μm diameter and 1000-μm length was connected to the soma. The axon began with a hillock of 40-μm length, which was reconstructed based on overall measurements from previously published RGC studies (Fohlmeister and Miller, 1997; Fried et al., 2009; Jeng et al., 2011). This was followed by an AIS region of 0.94-μm diameter and 40-μm length. A sufficient number of morphological compartments (>1000) were used for the axon to ensure accurate spatial granularity. All RGC model parameter settings can be found in Guo et al. (2016).

**FIGURE 1:**
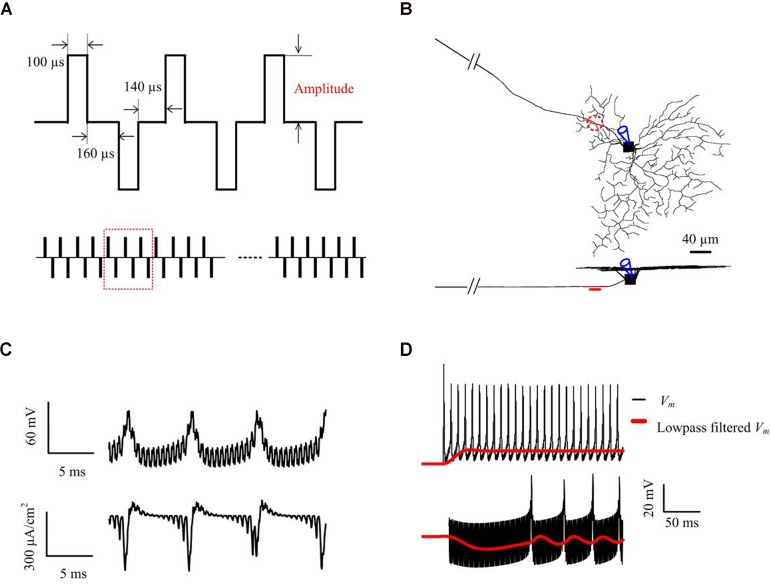
Computational simulations of 2-kHz HFS. **(A)** Applied constant-amplitude stimulation. Stimulus waveforms consisted of biphasic constant-current pulses with a pulse width of 100 μs per phase, and a 160-μs inter-phase interval. The anodal-cathodal inter-phase interval was set to 140 μs (Twyford et al., 2014). The stimulus train amplitude remained constant within each trial but varied across trials. **(B)** Stimulation and measuring locations in the RGC model shown in top- and side-views. The epiretinal stimulus electrode (shown as a red, dashed line, or disk) was placed 5-μm from the AIS center (red compartment) and spiking responses were obtained from the soma. **(C)** An example of simulated somatic membrane potential and total membrane ionic current traces. The stimulus artifact can be seen embedded within the somatic response. **(D)** An example of simulated somatic membrane potential (black) and low-pass filtered membrane potential (red), with 10 μA (upper panel) and 63 μA (lower panel) 2-kHz stimulation. Membrane potential *V_m_* was smoothed by a 3rd order Butterworth 20-Hz low-pass filter. This filtered membrane potential was used to examine the subthreshold (non-spiking) effects of HFS stimulus trains at different stimulus strengths.

For simulation of extracellular stimulation, we used a circular electrode disk in monopolar configuration. The extracellular potential *V* at each location was approximated using the following expression (Jeng et al., 2011; Tsai et al., 2012; Barriga-Rivera et al., 2017):

(1) $$V(r,\;z)=\frac{I_{o}\rho_{e}}{2\pi R}\arcsin\left(\frac{2R}{\sqrt{{\left(r-R\right)}^{2}+z^{2}}+\sqrt{{\left(r+R\right)}^{2}+z^{2}}}\right)$$

where *r* and *z* are the radial and axial distances, respectively, from the center of the disk for (*z* > 0), *R* is the radius of the disk (*R* = 15 μm), *I_o_* is the stimulus current, and ρ_*e*_ is the extracellular resistivity as described by Mueller and Grill (2013). The stimulus electrode was placed epiretinally 5 μm above the center of the AIS (Figure 1B), unless otherwise stated. RGC responses were recorded at the center of the somatic compartment (Figure 1C). The electrical stimulation waveforms and parameters were all adapted from previous HFS experimental studies (Cai et al., 2013; Twyford et al., 2014; Guo et al., 2018b) (see Figure 1A and *in vitro* methods for more details of stimulation parameters).

To examine the effects of HFS on the (non-spiking) RGC membrane polarization over time, we lowpass-filtered the membrane potential *V_m_* with a 3rd-order Butterworth filter with 20 Hz 3-dB cut-off frequency to remove spiking activities and the stimulus artifacts (e.g., see Figure 1D). Membrane depolarization and hyperpolarization induced by HFS was presented by the low-pass filtered membrane potential *V_m,LP_* normalized to the resting potential *V_rest_* i.e., *V_m,LP_-V_rest_*. All processing was performed either offline in Matlab (Mathworks Inc, Natick, MA, United States), or online within NEURON.

### Retinal Whole-Mount Preparation and Whole-Cell Patch Clamping

All procedures were approved by the UNSW Animal Care and Ethics Committee and were carried out in compliance with the Australian Code of Practice for the Care and Use of Animals for Scientific Purposes. Wild-type C57BL/6 female or male mice aged 4–8 weeks (purchased from Australian BioResource), were used in the *in vitro* experiments. Details on our whole-mount preparation and patch clamp recording with HFS can be found in Guo et al. (2018b) and Tsai et al. (2009). All elicited spikes were recorded at the soma after application of synaptic blockade, comprising a cocktail of synaptic blockers (in mM) consisting of 0.01 NBQX (2,3-Dioxo-6-nitro-1,2,3,4-tetrahydrobenzo[f]quinoxaline-7-sulfonamide) to block AMPA/kainate receptors, 0.05 D-AP5 [(2R)-amino-5-phosphonovaleric acid) to block NMDA receptors, 0.02 L-AP4 (L-(+)-2-Amino-4-phosphonobutyric acid] to block mGluR6, 0.1 picrotoxin (pic) to block GABAa/c receptors and 0.01 strychnine (stry) to block glycinergic receptors (Yang et al., 2018). The efficacy of the synaptic blockade was confirmed by the absence of RGC light responses.

We delivered HFS using a STG 4002 stimulator (MultiChannel Systems hardware and software, Reutlingen, Germany), with a stimulus duration of 300 ms. We began by stimulating the RGCs with conventional 2-kHz extracellular biphasic pulse trains to investigate RGC stimulus-response profiles. Electrical stimulation waveforms were adapted from Twyford et al. (2014) and Cai et al. (2013). The stimulus waveforms were charge-balanced biphasic with a pulse width of 100 μs per phase and an inter-phase interval of 160 μs. Stimulus amplitudes ranged from 5 to 120 μA, in 5-μA steps. In addition, stimulation frequencies of 1.0 and 8.33 kHz were chosen to test the influence of stimulation frequency in shaping RGC stimulus-strength-dependent properties. Stimulation waveforms were adapted from Guo et al. (2018b) (see Figure 7A). The timing resolution of our STG 4002 stimulator was 20 μs. each pulse width is 40 μs. We provided a range of frequencies (8.33, 6.25, 5.0, 4.17, 3.33, 2.5, 2.0, and 1.67-kHz) of stimulation to investigate the stimulus-frequency-dependency of RGCs. We believe a frequency range up to 8.33 kHz was reasonable in providing a sufficiently large stimulation parameter space for inhibiting RGC response, as well as reasonable stimulation efficacy. In all *in vitro* experiments, each pulse amplitude was delivered three times. The mean spike-stimulus curve was calculated for each cell. For each RGC, we defined a 3D Cartesian (*x, y, z*) coordinate system, with the soma as the origin, such that the upper surface of the RGC dendritic field was aligned in the *x*-*y* plane and the RGC axon was aligned with the *y*-axis. A platinum-iridium stimulating electrode of 12.5-μm radius was placed at location 0, 0, and -40 μm.

### Perfusion Solutions With Different Ionic Concentrations

To characterize the effects of Na^+^ reversal potentials on RGC responses to electrical stimulation, extracellular concentrations were custom-made (instead of using Ames’ solution) in order to adjust the Na^+^ concentration. There are three different [Na^+^] solutions. Chemical components and concentrations of each solution are listed in Table 1, along with the final Na^+^ concentrations and the calculated reversal potentials. In particular, the extracellular Na^+^ concentration in the first and second low Na^+^ solutions were adjusted to achieve custom Na^+^ reversal potentials. Na^+^ reversal potentials were calculated by the Goldman equation with [Na^+^]_i_ of 19.5 mM in our internal solution recipe.

(2) $$E_{Na}=\frac{RT}{F}\ln\left(\frac{{\left[Na^{+}\right]}_{o}}{{\left[Na^{+}\right]}_{i}}\right)$$

where *E_Na_* is the reversal potential of Na^+^, *R* is the ideal gas constant (8.314 J mol^-1^ K^-1^), *T* is the temperature in kelvin (307 K), *F* is Faraday’s constant (96485 C mol^-1^). The sequence in which the three solutions were applied was randomly set to avoid possible ordering effects.

**Table 1:** Extracellular concentration and estimated Na^+^ reversal potentials for various [Na^+^] solutions.

Chemicals	Control Na^+^ solution	1st low Na^+^ solution	2nd low Na^+^ solution
NaCl	117.41	73.90	43.93
NaHCO_3_	22.62	22.62	22.62
KCl	3.10	3.10	3.10
KH_ 2_PO_4_	0.57	0.57	0.57
MgSO_ 4_	1.24	1.24	1.24
CaCl_2_	1.52	1.52	1.52
Ascorbic acid	0.10	0.10	0.10
Glucose	11.09	12	12
Choline chloride	NA	43.05	73.02
**Na^+^ external concentration and estimated reversal potential**			
[Na^+^]_o_ (mM)	140.0	96.5	66.6
*E_Na_* (mV)	52	42	32


## Results

### Stimulus-Strength-Dependent RGC Responses to Constant-Amplitude 2-kHz HFS

**FIGURE 2:**
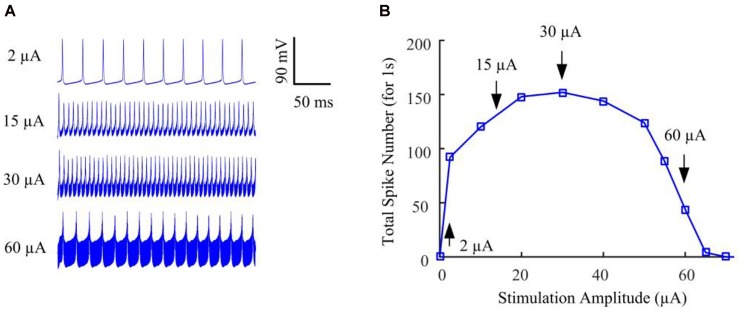
The RGC model exhibited strength-dependent responses to constant-amplitude 2-kHz HFS. **(A)** Somatic voltage recordings for stimulation amplitudes of 2, 15, 30, and 60 μA. Larger stimulation amplitude was accompanied by increasing stimulation artifacts. **(B)** We counted the number of evoked spikes at the soma over a range of stimulus amplitudes. At relatively low stimulus magnitudes (i.e., <30 μA), RGC spiking activity at the soma typically increased with stimulus amplitude. However, as the stimulus strength increased further (i.e., from 30 to 70 μA), the elicited spike count decreased substantially, creating a non-monotonic response profile as a function of stimulus amplitude. Arrows with index numbers correspond to the spiking profiles in panel **A**.

We began by stimulating the model RGC with conventional (fixed-amplitude) 2-kHz extracellular biphasic pulse trains (see Figure 1A). The amplitude of the stimulus train remained constant for a given trial but varied across trials ranging from 0 to 120 μA. For each stimulus amplitude, we counted the total number of spikes elicited during the 1-s stimulation period. We then determined the number of evoked spikes as a function of stimulus amplitude. Figure 2A shows the simulated membrane potential recorded at stimulation amplitudes of 2, 15, 30, and 60 μA. Elicited spikes were gradually inhibited when the stimulation amplitude was higher than 30 μA, until they were fully suppressed at 70 μA. Figure 2B shows spike-stimulus profiles at the soma of the RGC model. At relatively low stimulus magnitudes (i.e., <30 μA), the model RGC spike rate increased with stimulus amplitude. However, as the amplitude increased further (i.e., >30 μA), the number of elicited spikes decreased substantially, thereby creating a non-monotonic response profile as a function of stimulus amplitude, in agreement with recent *in vitro* studies (Cai et al., 2013; Twyford et al., 2014).

### Mechanisms Underlying Stimulus-Strength-Dependent Spike Suppression

To investigate possible mechanisms underlying the non-monotonic extracellular response, and how this could be influenced by neuronal biophysical properties, we performed several sets of follow-up simulations and *in vitro* experiments.

#### *In silico* Investigation on HFS-Induced Inhibition

**FIGURE 3:**
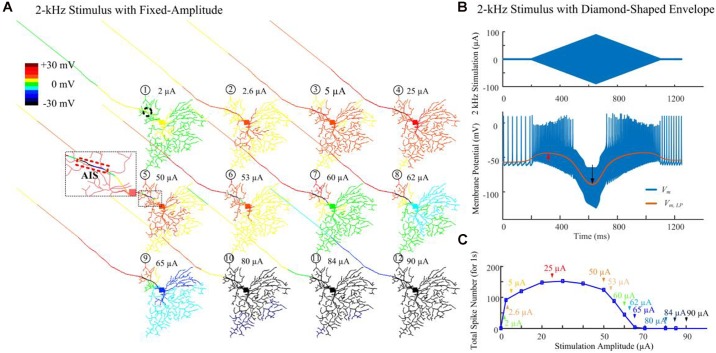
Modeled spike suppression using high-amplitude 2-kHz HFS. **(A)** The relative membrane depolarization and hyperpolarization (*V_m,LP_* – *V_rest_*) induced by constant-amplitude 2-kHz extracellular stimulation immediately following eighty stimulus pulses (40 ms from stimulus onset). Here the membrane potential changes were low-pass filtered to better represent the low-amplitude, long-duration changes during and after HFS. Small amplitude pulses (2 μA) only depolarized neurites near the electrode (dashed circle). When pulses of 50 μA were delivered, local hyperpolarization was apparent at the AIS and neighboring regions (indicated by the zoomed subplot) while the soma and dendrites were still depolarized (region with warm colors). The spatial extent of hyperpolarized regions progressively increased with higher stimulation amplitudes. **(B)** Transient HFS-induced membrane depolarization (red arrow) and hyperpolarization (black arrow) under 2 kHz diamond envelope stimulations (900 ms duration, 2 μA baseline with a peak of 90 μA). **(C)** Number of somatic spikes evoked with a 1-s, 2-kHz HFS over a range of stimulus amplitudes. Index numbers and arrows correspond to each subplot in panel **A**.

Figure 3A demonstrates modeled transient cell membrane voltage across the RGC’s dendritic arbor as the stimulus amplitude ranged from 0 to 90 μA for a 1-s duration 2-kHz HFS train shown in Figure 1A. We low-pass filtered the membrane potential values, henceforth denoted “slow potential,” to better visualize the small-amplitude, long-duration changes induced by the HFS. The slow potential over the entire neuron was examined 40 ms after stimulus onset (i.e., after eighty stimulation pulses). At this time, membrane potential had reached a steady state value. When a small-amplitude stimulus pulse train (2 μA) was delivered, the cellular membrane close to the stimulation site (the AIS region is indicated by the dashed rectangle) was depolarized (yellow). At the same time, a large portion of the peripheral dendritic branches were near their resting potential (green). When a HFS pulse train with 50-μA amplitude was delivered, local hyperpolarization was clearly evident near the AIS region (as indicated in the zoomed subplot). In addition, proximal neurites were depolarized. As the stimulus strength was increased further, distal neurites began to exhibit progressively stronger hyperpolarization. The index numbers correspond to the arrows in Figure 3C.

Figure 3B illustrates an example of slow potential transitions between depolarization (red arrow) and hyperpolarization (black arrow) at the soma during HFS. Here, a 2-kHz stimulus with diamond-shaped envelope (900 ms duration, 2 μA baseline with a peak of 90 μA) was used. With low stimulus amplitudes, the somatic membrane potential became increasingly depolarized (indicated by the red arrow in Figure 3B). However, with stronger pulses, membrane hyperpolarization was increasingly evident (indicated by the black arrow in Figure 3B).

**FIGURE 4:**
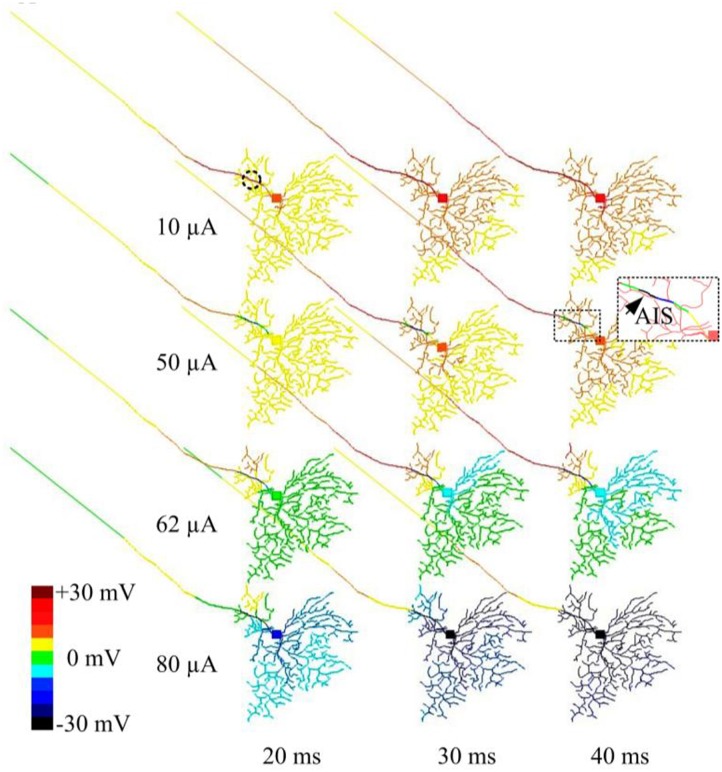
Modeled time-dependent membrane potential suppression with constant-amplitude 2-kHz HFS. The RGC model was stimulated by 10, 50, 62, and 80 μA 2 kHz HFS. At each stimulation level, baseline membrane potential at multiple time points (20, 30, and 40 ms after the stimulation onset) were obtained. At a stimulus amplitude of 10 μA, membrane potential across the whole cell was increasingly depolarized over time. At an amplitude of 50 μA, cumulative local membrane hyperpolarization (indicated by the arrow) was evident near the stimulation electrode (dashed circle). For stimulus pulses of 62 and 80 μA, most cellular regions were increasingly hyperpolarized.

In another set of simulations, time-dependent membrane behavior was examined when the RGC was stimulated by 2-kHz HFS pulse trains of 10, 50, 62, or 80 μA amplitude. The slow potentials across the entire cell were plotted at 20, 30, and 40 ms time points (at which steady state was reached) after stimulation onset (Figure 4). When 10-μA stimulus pulses were delivered, the membrane potential over the entire cell became increasingly depolarized over time. When stronger (e.g., 50 μA) stimulus pulses were delivered, hyperpolarization was evident in regions juxtaposing the stimulus electrode (indicated by the arrow and the cold colored regions in the zoomed subplot), while all other regions were depolarized. With even stronger stimulus pulses (e.g., 62 and 80 μA), most cellular regions became increasingly hyperpolarized over time. This HFS-induced hyperpolarization, when of sufficient strength, suppressed RGC excitation, contributing to the diminishing RGC spike rates with high-amplitude stimuli. During high-amplitude HFS, membrane potential does not completely recover from the hyperpolarization before the onset of each successive pulse in the train. The hyperpolarization thus increases over time with each successive pulse, eventually causing complete spike suppression.

#### Hyperpolarization Is Caused by Outward Currents at Neurites Near the Electrode

**FIGURE 5:**
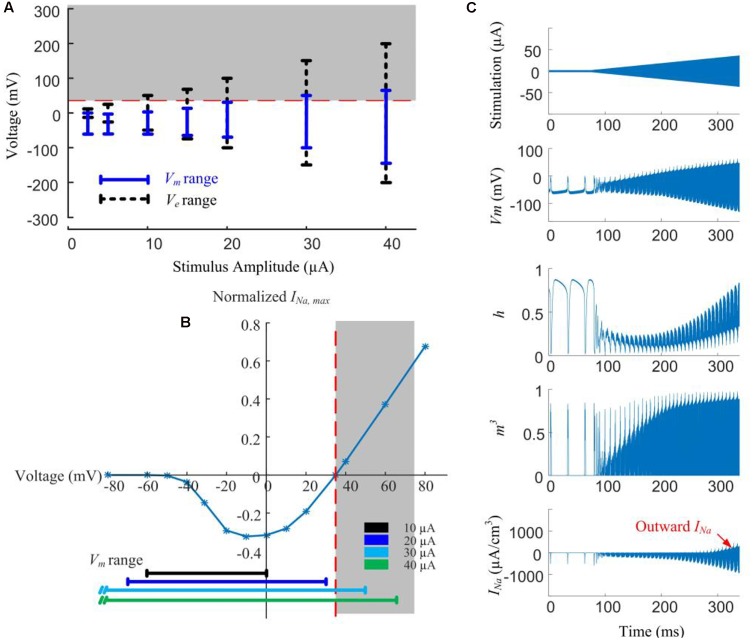
Modeled reversal of sodium current with high-amplitude HFS induces spike inhibition. **(A)** Range of extracellular voltage (*V_e_*) and transmembrane potential (*V_m_*) determined from voltage value differences at cathodal and anodal phase peaks. The shaded region indicates the voltage range (>35 mV) where reversal of sodium current occurs. **(B)** Normalized I-V curve of model RGC peak sodium current. Peak sodium current (*I_Na,max_*) becomes outward when *V_m_* is above its reversal potential of 35 mV (the shaded region). This reversal occurs when the stimulus amplitude is higher than 20 μA. **(C)** Reversal of sodium current during 2-kHz amplitude-modulated stimulation (250 ms duration, 2 μA baseline ramping to a peak of 40 μA). For HFS amplitudes higher than 30 μA, the activation (*m*) and inactivation (*h*) gating variables of the sodium current (neurites below the electrode) are entrained by the extracellular voltage changes. The sodium current becomes increasingly outward with higher HFS amplitudes (indicated in the bottom right panel).

In Figure 5A, local *V_e_* (extracellular voltage) and *V_m_* (transmembrane potential) are recorded below the stimulus electrode as a function of stimulus amplitude. Given low-amplitude stimuli (<10 μA), the intracellular membrane potential (*V_i_*) is the main contributor to *V_m_* due to the low magnitude of *V_e_*. However, with stimulus currents in excess of 20 μA, the magnitude of *V_e_* becomes more dominant and starts exceeding the sodium reversal potential, resulting in the *V_m_* range increasing in proportion to the increasing stimulus amplitude.

For a stimulus amplitude of 20 μA, *V_m_* spanned from -40 to +30 mV at the peaks of the cathodic and anodic phases, respectively. Because *V_m_* is always below the reversal potential of sodium (*V_Na_*) activation of sodium channels by the stimuli therefore causes depolarization, and subsequently, action potentials. At stimulus amplitudes >20 μA, *V_m_* begins to exceed *V_Na_* (Figure 5A, shaded region). Sodium channel activation under such conditions elicits an outward current (Figure 5B), causing hyperpolarization. The magnitude of the outward sodium current increases with increasing stimulus strength, hyperpolarizing the affected neurites, thus suppressing spike generation.

In Figure 5C, HFS with a ramped amplitude (top panel) is used to demonstrate the transient cell membrane behavior for stimulus amplitudes ramping from 2 to 40 μA. The sodium channel activation (*m*) and inactivation (*h*) gating variables start to passively following the extracellular voltage changes with increasing HFS amplitude. At the same time, the outward sodium current becomes increasingly stronger with higher HFS amplitudes (bottom right panel).

Our simulations shown in Figure 5 indicated that higher stimulation amplitudes can result in a change in the direction of the voltage-gated sodium current, such that the sodium current becomes increasingly outward.

#### The Stimulus-Strength-Dependent Response Profile Can Be Altered by Sodium Channel Properties

**FIGURE 6:**
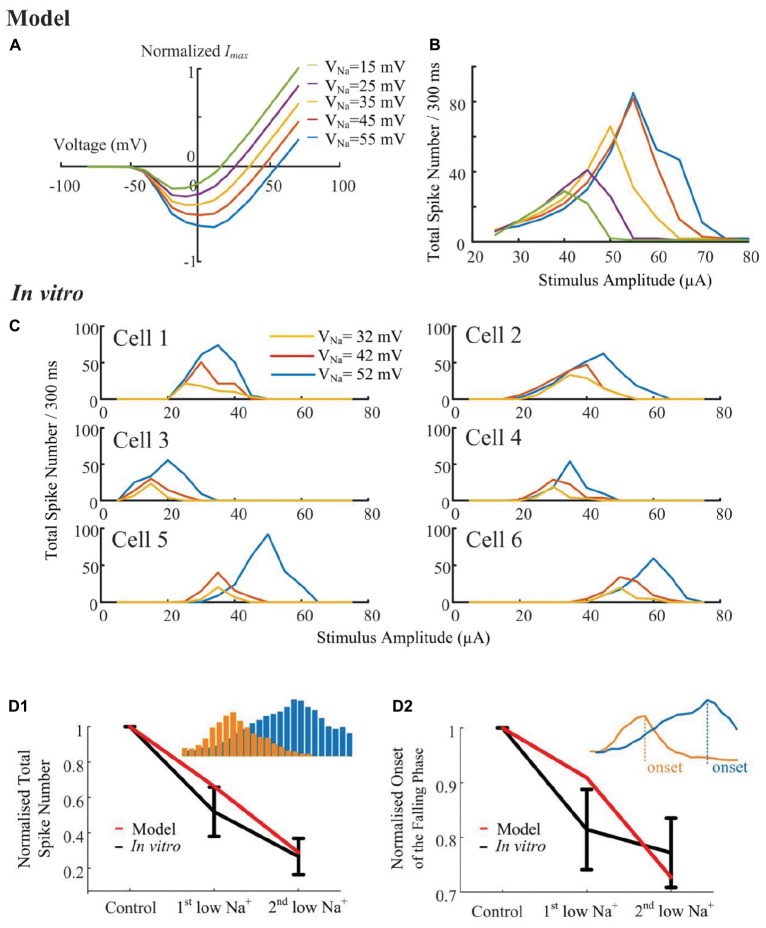
Sodium reversal potentials alter the strength-dependent response. **(A)** Normalized I-V relationship of the model RGC sodium current for various reversal potentials (*V_Na_*). Shifting *V_Na_* to a more positive value delays the reversal of the sodium current. **(B)** The modeled stimulus-response profile for various *V_Na_* values. Shifting *V_Na_* to a more positive value increases RGC excitability during HFS, postponing the suppressive effect, and *vice versa*. **(C)** *In vitro* results of HFS response curves with different *V_Na_* values (*N* = 6). The experimentally recorded RGC responses in mouse RGCs generally agree with the simulation results shown in panel B, with respect to the changes in amplitude and width of the response curve. **(D1,D2)** Comparison of model-prediction (red) and experimental data (black) in response to different Na^+^ solutions. Model predictions and *in vitro* data exhibited similar normalized trends of the total elicited spike number during all pulse trains (D1), and the normalized onset of the falling phase in the spike-stimulus curve in which the total spike numbers saturated or declined (D2). Examples of total elicited spike number and onset was provided in subplots in D1 and D2, respectively. The error bars indicate standard deviation.

According to the simulated sodium I-V relationship (Figure 6A), shifting the sodium reversal potential should advance or postpone the reversal of the sodium current, and consequently influence the non-monotonic response profile. To verify the hypothesis that the stimulus-strength-dependent response profile is influenced by *V_Na_*, we progressively altered the sodium (Figure 6B) reversal potential throughout the cell, while examining the elicited spikes in response to a range of HFS amplitudes. A stimulus electrode was positioned epiretinally 40-μm above the RGC soma. The *in silico* results suggested that the strength-dependent response profiles observed at the soma were progressively altered by changing the sodium reversal potential. When *V_Na_* was shifted to more negative values by changing extracellular sodium concentration, a marked decrease in the width and height of the response curve occurred (Figure 6B). Shifting the sodium reversal potential to more positive values resulted in the opposite effect. We also simulated the influence of other ionic currents such as *I_K_, I_Ca_, I_h_*, and *I_CaT_*. These currents did not significantly influence the response profile (see Supplementary Figure S1), suggesting the important role of sodium channel properties in shaping RGC responses to HFS.

To further validate the computational simulation results, the strength-dependent response profiles were also studied in *in vitro* experiments. In these experiments, we modified *V_Na_* by altering the extracellular [Na^+^] concentration. There are three different [Na^+^] solutions, with their chemical components and concentrations listed in Table 1, along with the final Na^+^ concentrations and calculated reversal potentials. In Figure 6C, despite the biological variance across RGCs, the total spikes evoked and the onset of spike suppression (i.e., stimulus amplitude associated with decreasing spike count) increased with increasing sodium reversal potential. In Figure 6D1,D2, normalized trends in HFS response curves are plotted as a function of [Na^+^] extracellular concentration. *In vitro* and modeled data demonstrated the same trends in the total elicited spike number during all pulse trains, as well as the onset of the falling phase in the spike-stimulus curve, in which the total spike numbers saturate or decline. Both our modeling and *in vitro* results suggested that voltage-gated sodium channel properties can strongly alter the shape of the stimulus-strength-dependent response profile.

#### Stimulus Induced Spike Inhibition Can Be Maximized With Sufficient Pacing Rate

**FIGURE 7:**
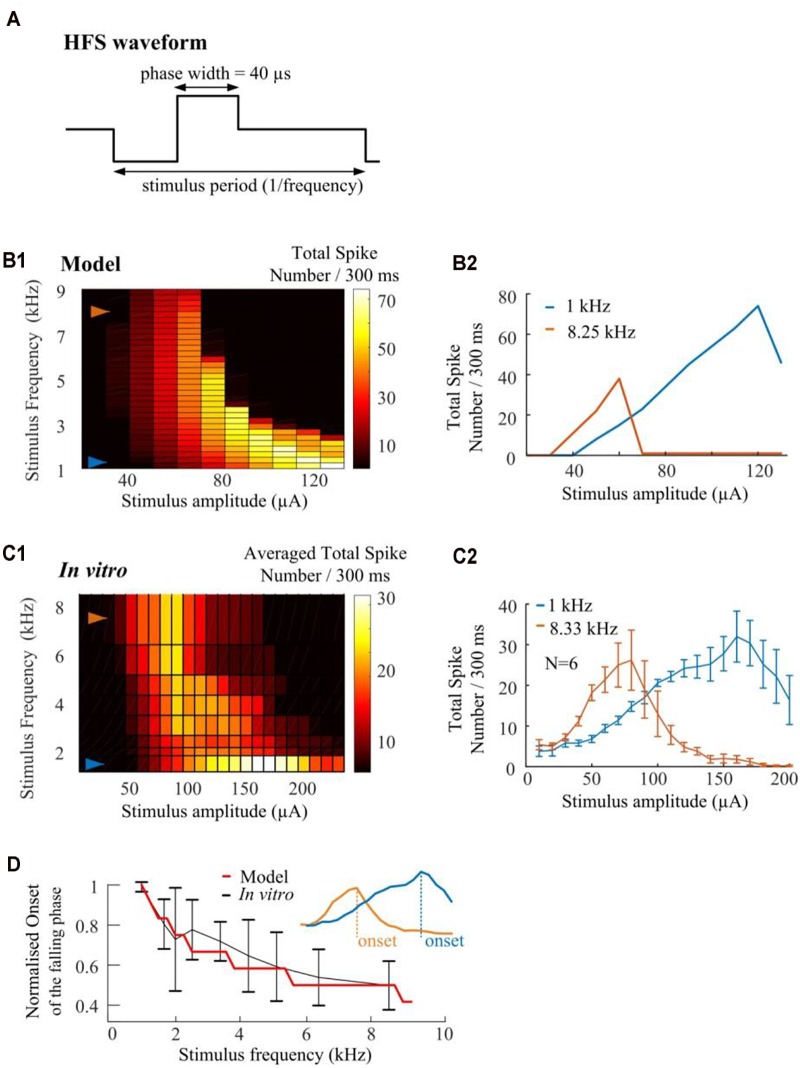
High frequency pulse trains require less amplitude to inhibit spikes in both *in silico* simulations and *in vitro* experiments. **(A)** Cathodic-first, charge-balanced biphasic stimuli with a pulse width of 40 μs per phase were used. Inter-pulse delay was set to be rate-specific (from 1.0 to 9.0 kHz). **(B1)** *In silico* prediction of stimulus-dependent (from 10 to 130 μA) RGC spike behavior in response to a range of stimulus frequencies. All pulse trains were 300 ms in duration. **(B2)** Modeled stimulus-dependent RGC spikes at 1.0 and 8.25 kHz as indicated by arrows in B1. **(C1)** Experimentally-recorded activation map showing the averaged total spike number elicited in six mouse RGCs in response to a range of stimulation amplitudes (from 10 to 240 μA) and frequencies (from 1.0 to 8.33 kHz). Each stimulation pulse train was delivered three times. The mean spike-stimulus curve was calculated for each cell and the overall mean was calculated again across all RGCs. **(C2)** Whole-cell recording of 1-kHz and 8.33-kHz stimulus-dependent RGC spikes for six mouse RGCs with standard error bars. **(D)** Comparison of model-predicted (red) and experimental data (black) in response to multiple stimulus frequencies. Modeled and *in vitro* results demonstrated similar frequency-dependent onset of the falling phase in the spike-stimulus curve. An example of onset was shown in the subplot. The error bars indicate standard deviation.

We conduct *in silico* investigations to explore the ability of HFS to suppress RGC spikes over a wide range of stimulus frequencies (1.0–9.0 kHz, in 0.25-kHz steps). We modified HFS waveforms to generate stimulus frequencies up to 9-kHz. Cathodic-first, charge-balanced biphasic stimuli with a pulse width of 40 μs per phase were used (Figure 7A). All pulse trains were 300 ms in duration. A stimulus electrode was positioned epiretinally 40-μm above the RGC soma. The simulated spikes were observed and counted at the soma. As seen in Figure 7B1, the model predicts that HFS-induced inhibition could be maximized by increasing HFS pulse train frequency. The RGC model exhibited an increased slope of the rising phase in the spike-stimulus curve (the phase in which spike counts increase with increasing stimulus current) and concomitantly, an earlier onset of the falling phase (in which the averaged total spike numbers saturate or decline). Examples of modeled stimulus-dependent RGC spikes at 1.0 and 8.25 kHz in Figure 7B2 further indicated the strong frequency-dependent inhibition.

The strength-dependent response profiles at various stimulation frequencies were also observed in *in vitro* experiments. A range of frequencies (6.25, 5.0, 4.17, 3.33, 2.5, 2.0, and 1.67-kHz) of stimulation were used to show the stimulus-frequency-dependent RGC response. Figure 7C1 shows the averaged (*N* = 6) stimulus-dependent response curves from 1.0 to 8.33 kHz. Each pulse train was delivered three times. The mean spike-stimulus curve was calculated for each cell and the overall mean was calculated again across all RGCs (*N* = 6). The standard error of mean (SEM) was calculated to estimate the variability of the estimated mean of population-based RGC spike rates. For comparison, *in vitro* data suggested frequency-dependent inhibition which highly agrees with model predictions (also see our examples of *in vitro* stimulus-dependent RGC spikes at 1.0 and 8.33 kHz in Figure 7C2).

In Figure 7D, normalized trends in the spike-stimulus curve are plotted as a function of stimulus frequency. *In vitro* and modeled data demonstrated the same trends in the onset of the falling phase (as indicated in the subplot) in the spike-stimulus curve. In summary, both our modeling and *in vitro* results suggested that other than stimulus-strength dependency, HFS-induced spike suppression is also highly frequency-dependent and can be maximized by modulating stimulation frequencies.

## Discussion and Conclusion

The non-monotonic stimulus-strength-dependent response has been previously reported in several retinal studies. Boinagrov et al. (2010) and Boinagrov et al. (2012) demonstrated the existence of an upper stimulus threshold using *in vitro* patch-clamp recording and a spherical model of the soma. In their studies, somatic responses were inhibited when the monophasic stimulation pulse was above a certain amplitude. They suggested that sodium current reversal was the primary reason for the inhibition. Rattay (2014) later conducted *in silico* investigations using a dendrite-soma-axon computational model to propose an anodal block phenomenon, in which an anodic surround of the focal cathodic pulse caused the nerve membrane on the outer wall of a pipette to become hyperpolarized due to current converging toward the tip of the electrode. This upper threshold phenomenon was further studied using charge-balanced biphasic pulses of various amplitude and phase duration (Meng et al., 2018), suggesting that an upper threshold in the soma of RGCs can block axonal excitation only under limited stimulation conditions. However, these studies used electrical stimulation with single monophasic or biphasic pulses. The precise mechanisms underlying HFS-induced strength-dependent activation remain unclear. In this study, we used *in silico* investigations to guide *in vitro* design. We explored the possible mechanisms underlying the HFS-induced non-monotonic spiking response as a function of stimulus amplitude, and how this feature is affected by RGC biophysical properties. Our results indicate that mechanisms underlying the measured strength-dependent response are multifaceted, namely: (1) localized membrane hyperpolarization is generated near the stimulus electrode and subsequently propagates to other neurites to suppress RGC excitation; (2) influence of sodium channel kinetics can strongly alter the shape of the stimulus-strength-dependent response profile; suggesting the important role of sodium channel properties in shaping RGC responses to HFS; (3) the inhibitory effect induced by electric stimulation can be maximized with sufficient stimulation rate. In addition, our results indicate that the non-monotonic RGC response to HFS does not arise through synaptic circuitry, since all *in vitro* results were observed after application of synaptic blockade.

### Other Possible Mechanisms Underlying the Non-monotonic Response Profile

Studies in the peripheral nervous system suggest that strong electrical stimulation may induce tonic membrane depolarization, keeping the channels in the inactivated state, thereby increasing activation threshold and preventing further spiking (Kilgore and Bhadra, 2004; Williamson and Andrews, 2005). Bianchi et al. (2012) also explored this “depolarization block” phenomenon during intracellular current injections using a computational model of CA1 pyramidal neurons. More recently, Kameneva et al. (2016) used this phenomenon to explain RGC spike inhibition induced by 2-kHz HFS. In our study, the depolarization block does contribute to RGC spike inhibition at the beginning of the falling phase in the spike-stimulus curve, in which the total spike numbers saturate (see the spike-stimulus curve between 25 and 53 μA in Figure 3A). However, at stimulus amplitudes where the total spike numbers begin to decline significantly (>60 μA in Figure 3A), progressively stronger hyperpolarization become dominant in inhibiting neuronal activation. Kameneva et al. hypothesized that the HFS-induced stimulus-response pattern is caused by the cell-specific potassium channel density and the size of the axonal sodium channel band. However, to our knowledge, there is no direct experimental evidence showing potassium channel distribution across different retinal neurons. Furthermore, an experimental study revealed that not all RGC types have unique axonal sodium channel band properties (Fried et al., 2009). Therefore, further studies are required to better understand the factors that shape the responses of functionally-distinct retinal neurons to HFS.

The second hypothesis is the inability of neurons to fully recover from their refractory period during HFS (Kilgore and Bhadra, 2004). This was, however, questioned as a possible mechanism underlying high-amplitude and high-frequency stimulations by previous modeling (Kilgore and Bhadra, 2004) and experimental studies (Bowman and McNeal, 1986), which showed that neurons are able to reliably maintain high spike rates during HFS. Since the refractory period is mainly controlled by sodium channel kinetics and our results suggested the importance of sodium channel properties in shaping RGC responses to HFS, we believe that refractory period of a neuron could contribute to HFS-induced RGC inhibition. This possibility, however, is likely dependent on the sodium channel subtype(s) expressed and their distribution in a particular neuronal type. Non-uniform distribution of variable voltage-gated sodium channels has been identified in mammalian RGCs (Fjell et al., 1997; Boiko et al., 2003; Van Wart et al., 2007; O’brien et al., 2008), and these sodium channels may respond differently to identical stimulus frequencies, due to their specific absolute and relative refectory periods. For example, Na(v) 1.2 has been reported to show a greater accumulation of inactivation at higher frequencies of stimulation than Na(v) 1.6 (Rush et al., 2005). Our *in vitro* results shown in Figure 6 and the results from others (Cai et al., 2011, 2013; Twyford et al., 2014; Guo et al., 2018b) demonstrate the variances of HFS-induced inhibition across different RGCs. Additional modeling studies using a wide range of sodium channel properties will be required to elucidate the mechanisms underlying these recorded variances. A comprehensive model capable of describing RGC intrinsic diversity and their characteristic response to HFS would be a major improvement in this field (Guo et al., 2014, 2018a; Kameneva et al., 2016).

### Improving the Quality of Electrical Stimulation

Without knowing how best to stimulate the retina, the vision quality elicited by retinal prosthetic devices will remain poor and unnatural. Previous *in vitro* and modeling studies indicated that appropriate HFS neuromodulation may elicit preferential excitation of different RGCs in a manner similar to RGC responses to light in a healthy retina, i.e., ON and OFF RGCs which respond with an increase in neural spiking activity to an increase or decrease in light intensity, respectively (Cai et al., 2013; Twyford et al., 2014; Guo et al., 2018b). In these studies, to preferentially activate one neuronal type without simultaneously producing substantial responses in another type, the cell types should have different non-monotonic stimulus-strength-dependent responses. Better understanding of mechanisms underlying stimulus-strength-dependent responses may shed light on more sophisticated stimulation strategies to improve the efficacy of retinal prosthetic devices. It remains to be seen if knowledge of RGC mechanisms can be used for practical stimulation strategy design in visual prostheses. For example, further *in vitro* and modeling studies are required to validate the reliability and generalizability of the non-monotonic nature of population-based RGC responses. Moreover, current modeling results are largely limited to somatic simulations, due to the lack of experimental data recorded in other neural processes. Recent modeling studies suggest that inhibition induced by electrical stimulation in the soma may not necessarily occur in the axon (Rattay, 2014; Meng et al., 2018). However, *in vivo* studies indicate that high-amplitude inhibition in the retina could occur at higher visual processing centers (Barriga-Rivera et al., 2017). In our future work, a computational model will be validated by experimental data recorded in RGC axons, to better study spike initiation and propagation. This updated model should shed further insights into the complex mechanisms responsible for HFS-induced inhibition.

### Summary

In this study, using previously optimized ionic channel distributions and kinetic parameters for each cellular region and incorporating detailed cell morphology, we were able to predict RGC strength-dependent stimulus response patterns observed experimentally. Our computational modeling approaches allowed us to investigate a wide range of biophysical properties and stimulation settings beyond those recorded in the initial biological dataset, guiding further experimental design. The electric field can be accurately described by mathematical formalisms, and the neurons can be “probed” at resolutions well beyond those achievable by today’s state-of-the-art biological techniques, furthering our understanding of the effects of novel stimulus strategies by simulating RGC stimulus-response profiles over a larger stimulation parameter space than have previously been explored.

## Ethics Statement

All procedures were approved by the UNSW Animal Care and Ethics Committee and were carried out in compliance with the Australian Code of Practice for the Care and Use of Animals for Scientific Purposes.

## Author Contributions

TG, DT, SF, SD, and NL conceived and designed the study. CY, TG, and DT performed the *in vitro* experiments. TG and DT performed the computational simulations. TG, DT, AAA, GS, JM, PT, SF, and NL analyzed the data. All the authors drafted the manuscript and read and approved of the final manuscript.

## Conflict of Interest Statement

The authors declare that the research was conducted in the absence of any commercial or financial relationships that could be construed as a potential conflict of interest.

## Supplementary Material

FIGURE S1 Simulated influence of multiple ionic channels in shaping RGC stimulus-strength-dependent properties. **(A)** delayed rectifier potassium channel (IK). **(B)** hyperpolarization-activated non-selective cationic current (Ih). **(C)** L-type Calcium channel (ICa). **(D)** low-threshold voltage-activated calcium current (ICaT). Conductance of each channel was set to be 50, 100, and 150% in the RGC model. Model settings and stimulation parameters are as same as in Figure 1.
