# Erratum: Mediating Retinal Ganglion Cell Spike Rates Using High-Frequency Electrical Stimulation

**DOI:** 10.3389/fnins.2019.00910

**Published:** 2019-08-28

**Authors:** Frontiers Production Office

**Affiliations:** Frontiers Media SA, Lausanne, Switzerland

**Keywords:** neuromodulation, retinal ganglion cell, high-frequency electrical stimulation, retinal implant, computational modeling, *in vitro* patch-clamp

Due to a production error, the captions within Figure 6C artwork have been incorrectly labeled as “blue (32 mV), red (42 mV), and orange (52 mV)” from top to bottom, instead of “orange (32 mV), red (42 mV), and blue (52 mV)”. The corrected Figure 6 appears below.

**Figure 6:**
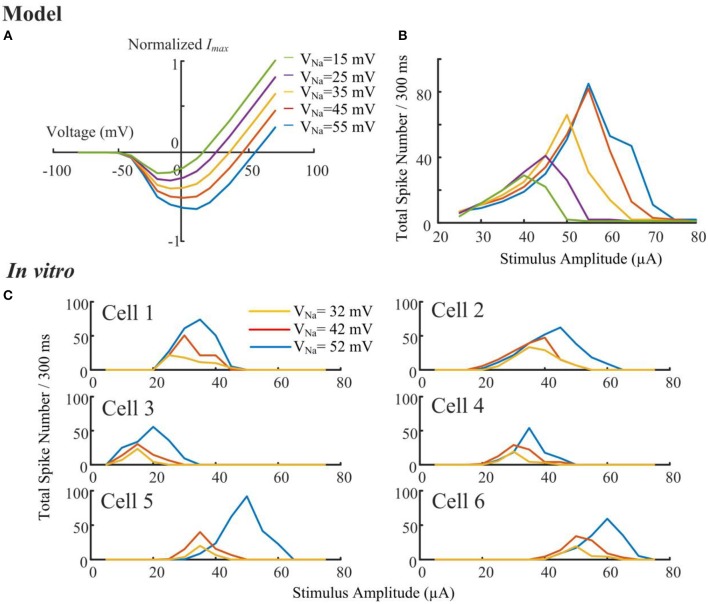
Sodium reversal potentials alter the strength-dependent response. **(A)** Normalized I-V relationship of the model RGC sodium current for various reversal potentials (*V*_*Na*_). Shifting *V*_*Na*_ to a more positive value delays the reversal of the sodium current. **(B)** The modeled stimulus-response profile for various *V*_*Na*_ values. Shifting *V*_*Na*_ to a more positive value increases RGC excitability during HFS, postponing the suppressive effect, and *vice versa*. **(C)** *In vitro* results of HFS response curves with different *V*_*Na*_ values (*N* = 6). The experimentally recorded RGC responses in mouse RGCs generally agree with the simulation results shown in panel B, with respect to the changes in amplitude and width of the response curve. **(D1,D2)** Comparison of model-prediction (red) and experimental data (black) in response to different Na^+^ solutions. Model predictions and *in vitro* data exhibited similar normalized trends of the total elicited spike number during all pulse trains (D1), and the normalized onset of the falling phase in the spike-stimulus curve in which the total spike numbers saturated or declined (D2). Examples of total elicited spike number and onset was provided in subplots in D1 and D2, respectively. The error bars indicate standard deviation.

The publisher apologizes for this mistake. The original article has been updated.

