# Supplementary figures and images for: Mediating Retinal Ganglion Cell Spike Rates Using High-Frequency Electrical Stimulation
Source: Front Neurosci. 2019 Apr 30;13:413. doi: 10.3389/fnins.2019.00413 (PMC6503046; doi:10.3389/fnins.2019.00413)

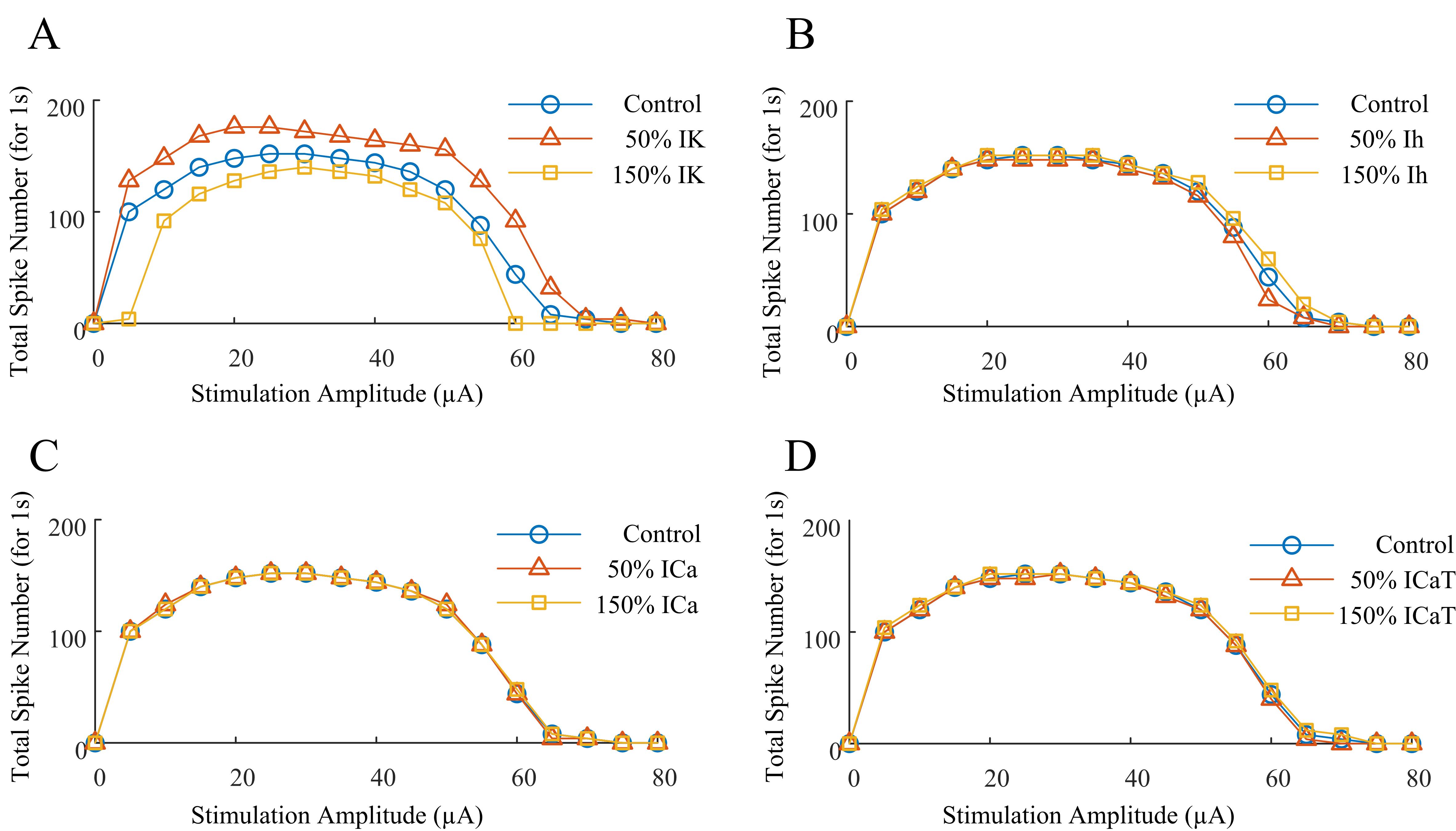

Supplement: FIGURE S1 — Simulated influence of multiple ionic channels in shaping RGC stimulus-strength-dependent properties. (A) delayed rectifier potassium channel (IK). (B) hyperpolarization-activated non-selective cationic current (Ih). (C) L-type Calcium channel (ICa). (D) low-threshold voltage-activated calcium current (ICaT). Conductance of each channel was set to be 50, 100, and 150% in the RGC model. Model settings and stimulation parameters are as same as in Figure 1. [file Image_1.JPEG]
